# Evaluation of Trueness and Precision in Extraoral 3D Facial Scanning Systems Using a 3D-Printed Head Model: An In Vitro Study

**DOI:** 10.3390/jcm14238384

**Published:** 2025-11-26

**Authors:** Viet Hoang, Tue Huu Nguyen, Trang Nhat Uyen Doan, Khue Minh Vu, Khang Chi Duong, An Sy Le, Lam Hung Tran, Phuc Ngoc Nguyen

**Affiliations:** 1 Department of Orthodontics and Pedodontics, Faculty of Dentistry, Van Lang University, Ho Chi Minh City 70000, Vietnam; viet.h@vlu.edu.vn; 2 Faculty of Dentistry, Van Lang University, Ho Chi Minh City 70000, Vietnam; nguyenhuutue123456@gmail.com (T.H.N.); trang.dnu03@gmail.com (T.N.U.D.); mnhkhue593@gmail.com (K.M.V.); duongchikhang2003@gmail.com (K.C.D.); lesyan.1610@gmail.com (A.S.L.); 3 Department of Prosthodontics, Faculty of Dentistry, Van Lang University, Ho Chi Minh City 70000, Vietnam; lam.th@vlu.edu.vn; 4 Department of Periodontology and Implantology, Faculty of Dentistry, Van Lang University, Ho Chi Minh City 70000, Vietnam

**Keywords:** 3D facial scan, trueness, precision, digital dentistry, photogrammetry

## Abstract

**Objective**: This in vitro study aimed to evaluate and compare the trueness and precision of four extraoral 3D facial scanning systems using a standardized 3D-printed human head model. **Methods**: A 3D-printed head model with 16 anatomical landmarks and 17 inter-landmark linear distances was fabricated using a high-resolution 3D printer. Caliper measurements were used as reference standards. The model was scanned 15 times by four systems: a handheld scanner (MetiSmile, Shining 3D, Hangzhou, China), a desktop scanner (RAYFace v2.0, Ray Co., Seongnam, Gyeonggi-do, Republic of Korea), and two mobile applications (Heges and Polycam, iPhone 15, Apple Inc., Cupertino, CA, USA). All digital distances were measured in Blender software. To assess intra-observer reliability, all measurements were repeated twice by the same examiner with a 3-week interval between sessions, and intra-class correlation coefficients were calculated using a two-way mixed-effects, single-measurement, absolute-agreement model (ICC 3,1). Trueness, defined as the absolute deviation from the reference caliper values, was compared across scanners using the Kruskal–Wallis test due to its non-normal distribution. Precision, regional trueness and precision values across the four scanners defined as the standard deviation of repeated scans, was analyzed using One-way ANOVA with Tukey post-hoc comparisons for normally distributed datasets (α = 0.05). Distances were measured digitally in Blender software, and trueness (absolute deviation from reference) and precision (standard deviation of repeated scans) were analyzed using the Kruskal–Wallis test and One-way ANOVA with Tukey post hoc comparisons (α = 0.05). **Results**: The Polycam application demonstrated the highest trueness (0.49 ± 0.32 mm), followed by MetiSmile (0.51 ± 0.36 mm), RAYFace (0.58 ± 0.39 mm), and Heges (0.73 ± 0.42 mm). The MetiSmile scanner showed the highest precision (0.12 ± 0.07 mm), while RAYFace and Polycam exhibited moderate precision (0.28 ± 0.19 mm and 0.15 ± 0.06 mm, respectively). Vertical measurements tended to be more accurate than horizontal ones, and the lower facial region showed smaller deviations; however, these differences were not statistically significant (*p* > 0.05). **Conclusions**: MetiSmile achieved the highest precision and Polycam the highest trueness. Although all systems showed mean deviations < 1 mm, only three demonstrated <0.6 mm accuracy (except for Heges scanner). These results suggest that professional and mobile-based scanners can provide clinically acceptable facial data for educational and preliminary digital workflow applications, though further validation under clinical conditions is required. This study provides quantitative evidence on the accuracy and repeatability of commonly available extraoral 3D facial scanning systems under controlled laboratory conditions. The results indicate that both professional-grade and mobile-based scanners can reproduce facial morphology with clinically acceptable deviations, particularly in flat and stable regions such as the forehead and chin. Although only three systems achieved mean trueness below 0.6 mm, all demonstrated errors within 1 mm, sufficient for diagnostic visualization, digital smile design, and preliminary virtual patient modeling. These findings support the safe and cost-effective adoption of extraoral facial scanning in dental education and treatment planning, while emphasizing the need for further validation in real clinical environments where motion, lighting, and soft-tissue variability may affect accuracy.

## 1. Introduction

The rapid advancement of digital technology has fundamentally transformed modern dentistry. What was once a supporting tool has now become an integral part of diagnosis, treatment planning, and clinical execution. The combination of intraoral scanning, computer-aided design and manufacturing (CAD/CAM), and three-dimensional (3D) facial scanning enables the creation of a “virtual patient,” allowing clinicians to visualize and simulate both hard and soft tissue structures with high accuracy and predictability. This integration is now essential across multiple disciplines, including orthodontics, prosthodontics, implantology, and maxillofacial surgery, where the relationship between dental structures and facial morphology is critical for esthetic and functional outcomes [1,2,3,4,5].

Among these digital innovations, 3D facial scanning plays a unique role as it captures the soft-tissue surface in three true dimensions. Compared with conventional two-dimensional photography or manual anthropometric measurement, which are time-consuming and operator-dependent, 3D facial scanning provides a fast, non-invasive, and reproducible alternative [6,7,8]. When integrated with intraoral scans or cone-beam computed tomography (CBCT) data, it supports digital workflows, enhancing diagnostic precision, virtual smile design, and postoperative assessment [9,10].

Various extraoral scanning systems are currently available, differing in technology, cost, and accessibility. Dedicated structured-light scanners, such as handheld or desktop systems, offer high accuracy but require substantial investment and controlled environments. In contrast, mobile-based applications using smartphone cameras provide portable and cost-effective alternatives but may be affected by lighting and operator variability [11,12,13,14]. Previous studies have shown that while smartphone-based photogrammetry achieves clinically acceptable deviations (<1 mm), its repeatability remains inferior to professional-grade systems [15].

However, despite growing adoption globally, the performance of these systems under standardized experimental conditions remains inconsistently reported. Many studies have evaluated selected devices but did not include a comparison of both professional and mobile-based scanners within the same experimental setup. Moreover, differences between vertical and horizontal measurements, as well as regional accuracy across facial thirds, are not fully understood. In Vietnam and other developing contexts, the high cost of professional scanners and the absence of benchmark data further limit clinical implementation.

Therefore, the study aimed to evaluate and compare the trueness and precision of four extraoral 3D facial scanning systems, a handheld scanner (MetiSmile), a desktop scanner (RAYFace v2.0), and two smartphone-based applications (Heges and Polycam), using a standardized 3D-printed human head model. By analyzing both overall and region-specific performance, this study seeks to provide objective evidence for selecting suitable extraoral scanning technologies and to promote the rational integration of digital facial data into contemporary dental workflows.

## 2. Materials and Methods

### 2.1. Study Design

The in vitro methodological study was designed to evaluate and compare the trueness and precision of four extraoral 3D facial scanning systems using a standardized 3D-printed human head model. All experiments were conducted in a controlled laboratory setting to minimize lighting, temperature, and operator variability. Ethical approval was not required as no human participants were involved.

A standardized 3D-printed human head model was used as the reference object for all measurements. Four extraoral 3D facial-scanning modalities were evaluated:

(1) A handheld structured-light scanner (MetiSmile, MetiSmile Technology Co., Ltd., Shenzhen, China),
(2) A desktop structured-light scanner (RAYFace v2.0, Ray Co., Ltd., Seoul, Republic of Korea),
(3) A smartphone-based LiDAR application (Heges v1.7.2, Heges Technologies Inc., San Francisco, CA, USA), and
(4) A smartphone-based photogrammetry application (Polycam, Polycam Inc., San Francisco, CA, USA).

Each system performed 15 independent scans of the head model. All digital scans were processed in Blender software, where inter-landmark linear distances were extracted. Reference measurements were obtained using a digital vernier caliper, allowing comparison of trueness and precision across all scanning modalities.

### 2.2. Human Head Model Design

A 3D-printed human head model was designed and conceptualized using Blender software (v4.5.2; Blender Foundation, Amsterdam, The Netherlands), based on the “MetaHuman Head Reference” dataset educational and non-commercial license [16]. The model dimensions were standardized according to anthropometric data of adult Asian individuals, with a mean head circumference of 56 cm [17]. Corresponding design coordinates in the software were defined along the transverse and vertical axes as follows: X = 23.66 cm, Y = 23.26 cm, and Z = 36.55 cm.

**Figure 1:**
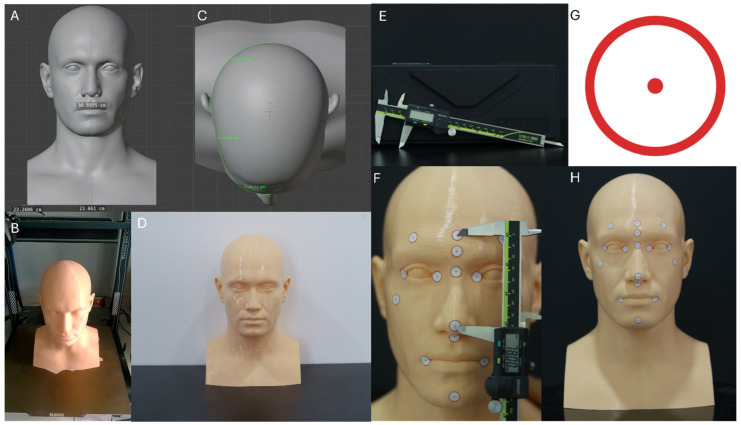
Workflow for head model design. (**A**) Model design using Blender software (version 4.5.2; Blender Foundation, Amsterdam, The Netherlands); (**B**) Completion of 3D printing process; (**C**) Standardized head circumference of 56 cm; (**D**) Final printed head model. (**E**) Digital vernier caliper (Mitutoyo Corp., Kawasaki, Japan); (**F**) Measurement of anatomical landmarks. (**G**) Landmark design using Canva software v1.109.0 (Canva Pty Ltd., Sydney, Australia); (**H**) 3D-printed head model after landmark placement.

The model was fabricated using fused deposition modeling (FDM) technology with a skin-tone PETG filament. After printing, basic post-processing included removal of support structures and light mechanical smoothing of the surface. A thin matte spray coating was applied, when necessary, to further reduce gloss and minimize surface reflections that could affect scanning accuracy (Figure 1).

Printing parameters were as follows:

- Printer type: Anycubic Kobra 3 (FDM) (Anycubic, Shenzhen, China)
- Layer height: 0.05 mm (high-detail setting)
- XY resolution: ~100 µm (0.4 mm nozzle)
- Material: Skin-tone PETG filament (Anycubic PETG Skin, 1.75 mm diameter, Anycubic, Shenzhen, China)
- Rationale: PETG provides dimensional stability, low warpage, and a naturally matte, non-reflective surface that is suitable for optical scanning.

### 2.3. Scanning Protocols

A 3D-printed human head model was positioned on a table at a height of 1 m. The experiment was conducted in a closed room of 15 m^2^ with white walls, an ambient illumination of 1000 lux, a temperature of 24 ± 2 °C, and an atmospheric pressure of 760 mmHg.

**Figure 2:**
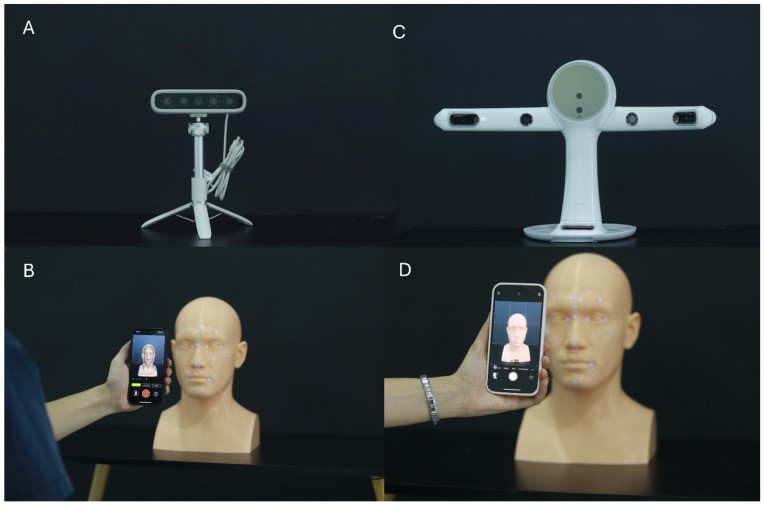
Four three-dimensional facial scanning systems. (**A**) Handheld facial scanner MetiSmile (Shining 3D Tech Co., Hangzhou, China); (**B**) Desktop facial scanner RAYFace (RAYFace v2.0; Ray Co., Ltd., Seongnam, Gyeonggi-do, Republic of Korea); (**C**) Mobile facial scanning application on iPhone 15 (Apple Inc., Cupertino, CA, USA) using Heges (Marek Simonik, Ostrava, Czech Republic); (**D**) Mobile facial scanning application on iPhone 15 (Apple Inc., Cupertino, CA, USA) using Polycam (Polycam Inc., San Francisco, CA, USA).

Four extraoral 3D facial-scanning systems were used in this study, each following a standardized acquisition protocol (Figure 2). The MetiSmile handheld scanner operated at a distance of 40–50 cm under neutral indoor lighting (~500 lux), capturing one continuous 360° sweep over 15–20 s. The RAYFace v2.0 desktop system scanned the model at a fixed 50 cm distance using five synchronized cameras under controlled 5500 K shadow-free lighting, generating three automatically merged composite scans. The Heges LiDAR-based iOS application was used at 30–40 cm in diffuse ambient light and acquired three circumferential passes (frontal, right, and left) to reduce occlusion. The Polycam photogrammetry mode collected 80–120 overlapping images around the model at 30–50 cm under evenly distributed 5000 K lighting before reconstructing the final mesh. Each modality produced 15 independent datasets for analysis (Table 1).

### 2.4. Landmark Placement and Reference Measurements

Sixteen anatomical facial landmarks were identified on the 3D-printed head model and marked using standardized 1 cm circular adhesive stickers featuring a printed red centroid. These landmarks included:

1. Central Forehead (CF)
2. Right Frontal (RF)
3. Left Frontal (LF)
4. Glabella (Gb)
5. Nasion (N)
6. Pronasale (Prn)
7. Subnasale (Sn)
8. Right Exocanthion (ExR)
9. Left Exocanthion (ExL)
10. Right Endocanthion (EnR)
11. Left Endocanthion (EnL)
12. Right Cheilion (RCH)
13. Left Cheilion (LCH)
14. Pogonion (PoG)
15. Right Zygion (ZR)
16. Left Zygion (ZL)

A total of 17 inter-landmark linear distances were measured between the following pairs of anatomical points: RF–CF, LF–CF, CF–Gb, CF–PoG, ExR–ExL, EnR–EnL, ExR–EnR, EnL–ExL, Gb–PoG, Gb–N, N–Prn, Prn–Sn, RCH–LCH, Sn–PoG, RCH–PoG, LCH–PoG, and ZL–ZR. These distances collectively represented upper, middle, and lower facial regions.

Landmark placement was performed by a single calibrated operator and verified independently by a second examiner using high-resolution photographs superimposed onto Blender reference coordinates. This verification process yielded a mean placement deviation of 0.22 ± 0.06 mm, confirming accurate and reproducible landmark localization. The center of each marker was defined as the geometric centroid of the printed circle to ensure consistency across caliper and digital measurements.

Reference physical measurements were obtained using a digital vernier caliper (Mitutoyo Model 500-181-30(N), Kawasaki, Japan) with a measurement range of 0–150 mm, resolution of 0.01 mm, and accuracy of ±0.02 mm. Each of the 17 distances was measured ten times by the same trained operator to minimize measurement bias. Intra-operator reliability, assessed using the intraclass correlation coefficient (ICC), demonstrated excellent repeatability (ICC = 0.998, 95% Confidence Interval: 0.996–0.999).

### 2.5. Data Analysis

Statistical analyses were performed using SPSS version 25.0 (IBM Corp., Armonk, NY, USA). All inter-landmark linear distances extracted from the four scanning systems were compared against the corresponding reference measurements obtained from the digital vernier caliper. Data distribution for each distance–scanner combination was evaluated using the Shapiro–Wilk test. Homogeneity of variances was assessed using Levene’s test.

Definitions (ISO 5725-1:1994; Accuracy (trueness and precision) of measurement methods and results—Part 1: General principles and definitions. International Organization for Standardization: Geneva, Switzerland, 1994.)

1. Trueness was defined as the absolute deviation between the mean digital measurement of each scanner and the corresponding caliper reference value.
2. Precision was defined as the standard deviation (SD) of the repeated measurements within each scanner for each inter-landmark distance.

Shapiro–Wilk testing demonstrated that trueness values did not follow a normal distribution, as deviation-based metrics tend to be positively skewed across scanners. Because the normality assumption was violated, the Kruskal–Wallis test was applied to compare trueness across the four scanning systems, providing a suitable non-parametric alternative for non-normal repeated-measures data.

For normally distributed datasets, differences in precision across the four scanners and trueness and precision values for each anatomical region were evaluated using One-way Analysis of Variance (ANOVA), followed by Tukey’s Honestly Significant Difference (HSD) post hoc test to determine pairwise significance. For non-normal datasets, the Kruskal–Wallis test was applied as a non-parametric alternative to compare trueness across scanners. A significance level of α = 0.05 was used for all statistical tests. The dataset had a nested and correlated structure (17 distances × 4 scanners × repeated scans), which theoretically favors a linear mixed-effects model with the scanning system as a fixed effect and anatomical landmark as a random effect. However, given the exploratory nature of this in vitro pilot study and the limited number of repeated observations per landmark, simplified ANOVA and Kruskal–Wallis tests were applied to provide transparent and interpretable comparisons across systems. Future studies with larger sample sizes will incorporate mixed-effects modeling to fully account for hierarchical variance.

## 3. Results

### 3.1. Trueness and Precision Across Scanning Systems

The trueness values were obtained from the four scanning systems. Polycam demonstrated the highest trueness (0.49 ± 0.32 mm), followed by MetiSmile (0.51 ± 0.36 mm) and RAYFace (0.58 ± 0.39 mm). In contrast, the Heges application showed the lowest trueness (0.73 ± 0.42 mm). The Kruskal–Wallis test was performed only on Polycam, MetiSmile, and RAYFace, as the Heges dataset exhibited extreme deviation levels that were not comparable to the other scanners, making joint non-parametric comparison inappropriate. The test yielded *p* = 0.148 (>0.05), indicating no statistically significant difference in trueness among these three systems. Because Heges exceeded the clinically acceptable threshold, its trueness results were reported descriptively but excluded from the Kruskal–Wallis test comparison (Table 2).

In terms of precision, MetiSmile achieved the greatest repeatability with 0.12 ± 0.07 mm. Polycam followed with 0.15 ± 0.06 mm, while RAYFace (0.28 ± 0.19 mm) and Heges (0.41 ± 0.17 mm) showed progressively lower precision. One-way ANOVA confirmed statistically significant differences across systems (*p* < 0.001), and Tukey HSD post hoc tests identified significant pairwise differences (Table 2).

### 3.2. Landmark-Based Trueness and Precision Analysis

Polycam and MetiSmile consistently produced the smallest deviations from the caliper reference, with most trueness values falling below 0.6 mm. These two systems maintained relatively stable accuracy across multiple regions, particularly for distances involving midline or flatter surfaces (e.g., RF–CF, LF–CF, CF–Gb).

RAYFace demonstrated moderate trueness, with deviations generally clustered between 0.6 and 1.0 mm. Although it performed comparably to Polycam and MetiSmile in several landmarks, increased deviations were noted in areas associated with curved or recessed anatomy such as ExR–EnR, EnL–ExL, and CF–PoG.

In contrast, Heges exhibited the highest trueness deviations, frequently exceeding 1.0 mm and showing the largest errors at landmarks with complex geometry. Notable examples include Prn–Sn, ExR–EnR, and RCH–LCH, where deviations approached or exceeded 1.5–1.8 mm. These findings indicate that Heges is more affected by surface curvature and shadow-prone regions than the other systems.

The precision plot likewise showed a consistent ranking across devices. MetiSmile achieved the highest repeatability, with precision values consistently below 0.15 mm for all 17 distances. This stable performance was observed across both straightforward (e.g., RF–CF, CF–Gb) and more complex distances (e.g., ExR–EnR, RCH–PoG), confirming uniform reproducibility.

Polycam displayed moderate precision, with standard deviations generally ranging from 0.15 to 0.30 mm. Although less consistent than MetiSmile, its repeatability remained within an acceptable range for most distances. RAYFace exhibited greater dispersion, showing variable precision across landmarks (approximately 0.3–0.6 mm), particularly in areas influenced by single-shot acquisition constraints.

**Figure 3:**
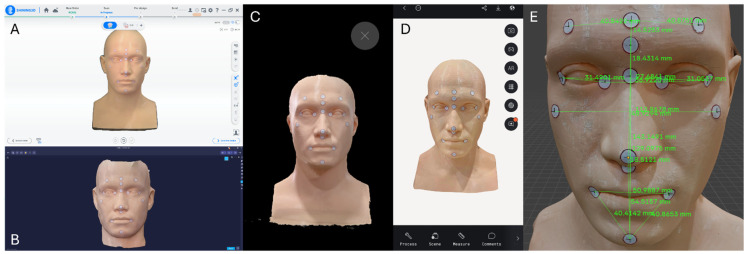
Four facial scanning systems correspond to measurement results. (**A**) Handheld facial scanner MetiSmile (Shining 3D Tech Co., Hangzhou, China); (**B**) Desktop facial scanner RAYFace (RAYFace v2.0; Ray Co., Ltd., Gyeonggi-do, Republic of Korea); (**C**) Mobile facial scanning application Heges (Marek Simonik, Ostrava, Czech Republic). (**D**) Mo-bile facial scanning application Polycam (Polycam Inc., San Francisco, CA, USA). (**E**) Measure the results on Blender software.

**Figure 4:**
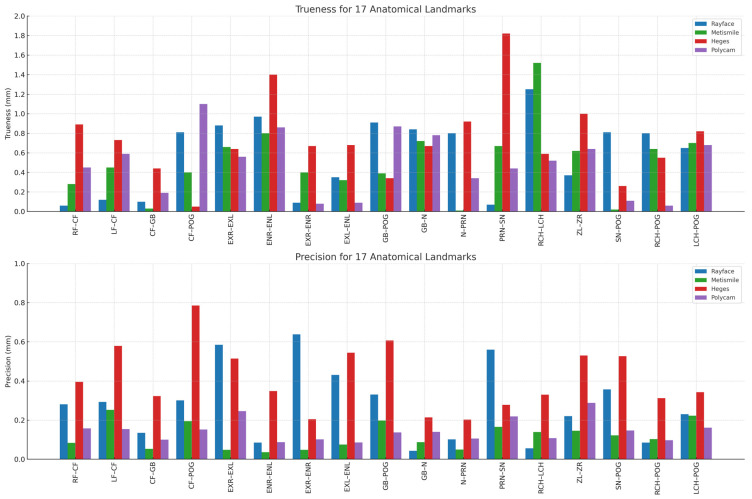
Trueness and precision values across 17 inter-landmark distances for the four 3D facial scanning systems. The upper panel illustrates the trueness (mm) of RAYFace, MetiSmile, Heges, and Polycam across all 17 anatomical distances using digital caliper measurements as the reference standard. Polycam and MetiSmile demonstrated the smallest deviations from the ground truth, whereas Heges consistently exhibited the largest trueness errors, particularly in landmarks with complex surface curvature. RAYFace showed moderate performance with deviations clustering between 0.6 and 1.0 mm. The lower panel displays the precision (mm) for all systems based on repeated scans. MetiSmile demonstrated the highest repeatability, with precision values consistently below 0.15 mm for all landmarks. Polycam showed moderate repeatability, RAYFace presented greater variability (approximately 0.3–0.6 mm), and Heges exhibited the lowest precision with several landmarks exceeding 0.6–0.8 mm. Together, the charts illustrate consistent performance differences among the systems, with structured-light and photogrammetry-based devices outperforming the mobile LiDAR application in both trueness and precision. Central Forehead (CF), Right Frontal (RF), Left Frontal (LF), Glabella (Gb), Nasion (N), Pronasale (Prn), Subnasale (Sn), Right Exocanthion (ExR), Left Exocanthion (ExL), Right Endocanthion (EnR), Left Endocanthion (EnL), Right Cheilion (RCH), Left Cheilion (LCH), Pogonion (PoG), Right Zygion (ZR), Left Zygion (ZL).

Finally, Heges demonstrated the lowest overall precision, with several landmarks exceeding 0.6–0.8 mm in standard deviation. The largest inconsistencies were observed in regions requiring detailed depth capture, such as ExR–EnR, ZL–ZR, and RCH–PoG. These results indicate that repeated measurements with Heges yield substantial variability, particularly in areas where LiDAR sensors struggle with depth resolution or lighting sensitivity (Figure 3). And below is the chart that illustrates the trueness and precision values for all 17 inter-landmark distances across the four facial-scanning systems. The figure highlights the variation in measurement performance among the devices, showing how each scanner differs in both accuracy components at specific anatomical regions. This visualization allows for a clear comparison of landmark-based deviations, revealing areas where certain scanners achieve higher trueness or greater precision than others (Figure 4).

### 3.3. Trueness and Precision by Facial Region

In the forehead region, the RAYFace and MetiSmile scanners demonstrated the highest trueness, with mean deviations of 0.27 ± 0.36 mm and 0.29 ± 0.19 mm, respectively. The two smartphone-based applications, Heges and Polycam, showed larger deviations of 0.53 ± 0.37 mm and 0.58 ± 0.38 mm, respectively. In the eye region, the Polycam application achieved the highest trueness (0.40 ± 0.38 mm), followed by MetiSmile (0.55 ± 0.22 mm) and RAYFace (0.57 ± 0.42 mm), while Heges presented the greatest deviation (0.85 ± 0.37 mm). For the nasal region, MetiSmile again demonstrated the best trueness (0.45 ± 0.33 mm), followed by Polycam (0.61 ± 0.26 mm), RAYFace (0.65 ± 0.39 mm), and Heges (0.94 ± 0.63 mm). In the cheek region, Polycam produced the most accurate results, with a mean deviation of 0.58 ± 0.09 mm, while the other devices showed increasing deviations: Heges (0.80 ± 0.29 mm), RAYFace (0.81 ± 0.62 mm), and MetiSmile (1.07 ± 0.64 mm). In the chin region, Polycam also yielded the smallest deviation (0.28 ± 0.34 mm), followed by MetiSmile (0.45 ± 0.38 mm), Heges (0.54 ± 0.28 mm), and RAYFace (0.75 ± 0.09 mm) (Table 3).

Regarding precision, the MetiSmile handheld scanner demonstrated the best repeatability, with the smallest standard deviations across nearly all regions. In the forehead region, MetiSmile showed the lowest variation (0.11 ± 0.06 mm), followed by Polycam (0.14 ± 0.07 mm), RAYFace (0.25 ± 0.12 mm), and Heges (0.39 ± 0.17 mm). Similar patterns were observed in the eye region (MetiSmile: 0.13 ± 0.08 mm; Polycam: 0.16 ± 0.06 mm; RAYFace: 0.32 ± 0.15 mm; Heges: 0.44 ± 0.16 mm) and in the nasal region (MetiSmile: 0.14 ± 0.09 mm; Polycam: 0.18 ± 0.07 mm; RAYFace: 0.34 ± 0.14 mm; Heges: 0.47 ± 0.19 mm). In the cheek region, MetiSmile again showed the highest precision (0.12 ± 0.07 mm), followed by Polycam (0.15 ± 0.05 mm), RAYFace (0.26 ± 0.11 mm), and Heges (0.41 ± 0.15 mm). Finally, in the chin region, MetiSmile maintained the greatest stability (0.10 ± 0.06 mm), followed by Polycam (0.13 ± 0.05 mm), RAYFace (0.30 ± 0.13 mm), and Heges (0.36 ± 0.14 mm) (Table 3).

## 4. Discussion

This in vitro study compared the trueness and precision of four extraoral 3D facial-scanning systems using a standardized 3D-printed head model. All systems produced sub-millimeter deviations, although their performance varied substantially. Three systems—MetiSmile, RAYFace, and Polycam—achieved mean trueness values below 0.6 mm, whereas the Heges LiDAR-based application showed noticeably higher deviations. These results align with previous reports indicating that professional structured-light scanners and photogrammetry-based applications generally outperform mobile LiDAR tools in quantitative facial surface reproduction [18,19,20].

Performance differences arise mainly from data-acquisition algorithms. Polycam’s photogrammetry enhances trueness by combining high-resolution 2D images but is susceptible to lighting variations. MetiSmile’s structured-light sensors yield superior precision due to real-time calibration and multi-camera triangulation. RAYFace prioritizes speed, leading to fewer depth points and moderate precision. Heges, reliant on mobile LiDAR, shows lower consistency because of short baseline and ambient light sensitivity. Among the tested systems, Polycam demonstrated the highest trueness (0.49 ± 0.32 mm). This can be attributed to its photogrammetric reconstruction pipeline, which combines a large number of overlapping high-resolution images to increase point-cloud density and reduce alignment errors. Polycam’s accuracy depends primarily on multi-view image acquisition and stable lighting. Pellitteri et al. similarly reported that photogrammetry-based applications can achieve clinically acceptable trueness when capture conditions are optimized [18].

MetiSmile exhibited the highest precision (0.12 ± 0.07 mm), reflecting consistent depth reconstruction across repeated scans. Its multi-camera structured-light architecture and continuous scanning trajectory likely minimize stitching artifacts and operator-dependent variation. Similarly, Bor et al. [19] identified handheld structured-light scanners as among the most reliable systems for quantitative soft-tissue capture.

RAYFace showed moderate trueness and precision, consistent with its rapid single-shot acquisition strategy, which favors scanning speed but generates fewer geometric data points than continuous structured-light approaches [21,22,23]. Heges showed the lowest trueness and precision among all systems, a finding consistent with prior research demonstrating the inherent limitations of mobile LiDAR technology, including lower point density, reduced depth accuracy, and greater susceptibility to ambient lighting fluctuations [24]. Although Heges was able to reproduce overall facial shape, its variability indicates that it is less suitable for applications requiring stable quantitative accuracy.

Region-specific patterns observed in this study showed that deviations tended to be smaller in flatter areas such as the forehead and chin, and larger in regions with complex concave–convex geometry such as the eyes, nose, and cheeks. These patterns reflect known challenges in 3D scanning, where surface curvature and occlusion can reduce measurement accuracy across all systems. However, no statistically significant differences were found among anatomical regions (*p* > 0.05), and therefore these observations represent trends rather than confirmed regional effects. Moreover, vertical measurements are anchored on stable midline landmarks (nasion, subnasale, pogonion) that align with the gravitational axis and are easily captured by all systems. In contrast, horizontal distances cross curved surfaces, increasing optical distortion and interpolation errors during point-cloud reconstruction, which may explain the higher deviations observed.

The present findings are broadly consistent with earlier investigations reporting that structured-light systems generally exhibit higher precision, while photogrammetry demonstrates competitive trueness under controlled conditions [18]. Smartphone-based LiDAR solutions, although accessible and convenient, remain limited by hardware constraints and environmental sensitivity, resulting in the lower measurement reliability observed here [24].

This study used 16 anatomical landmarks and 17 linear distances derived from 15 repeated scans per device, yielding a total of 1020 digital measurements. Although this design ensured measurement consistency, the study had several limitations. First, only one 3D-printed head model was used, limiting generalizability across different anatomical shapes and textures. Second, the landmark stickers had a diameter of 1 cm, which may introduce centroid uncertainties despite verification procedures. Third, all measurements and scans were performed by a single operator; although intra-operator reliability was high, inter-operator variability was not assessed. Fourth, Blender was used for digital measurement without external metrology validation beyond our pilot calibration. Fifth, the dataset has a hierarchical structure (17 distances × 4 systems × repeated scans), and although ANOVA and Kruskal–Wallis tests were used for exploratory analysis, a mixed-effects model would have better accounted for nested variability. Finally, this in vitro design does not reflect clinical factors such as involuntary motion, skin reflectivity, or inconsistent lighting, all of which may worsen real-world accuracy.

Clinical experience also shows that facial scanning is increasingly important when digital planning needs to be guided by the patient’s real soft-tissue appearance [25,26,27]. Hoang et al. used facial data to evaluate gingival display and design a precise digital surgical guide for a severe gummy-smile case, helping align the outcome with the patient’s esthetics [28]. Likewise, Nguyen et al. demonstrated that full-arch implant rehabilitation benefits from facial-guided positioning to achieve a restoration that fits naturally with the patient’s facial profile [29]. These examples highlight how accurate facial scanning and digital applications can support more predictable, precision and facially driven clinical decision-making and in the future, more comprehensive in vivo studies will be essential to validate these findings under real clinical conditions and to better understand the performance of each scanning modality in everyday practice [30].

## 5. Conclusions

This in vitro study quantitatively compared the trueness and precision of four extraoral 3D facial-scanning systems using a standardized 3D-printed head model. All scanners demonstrated sub-millimeter deviations, although their performance levels varied. Polycam achieved the highest trueness, MetiSmile showed the greatest precision, and Heges exhibited the lowest overall accuracy and repeatability. RAYFace demonstrated intermediate performance consistent with its single-shot structured-light design.

Landmark-based analysis revealed that flatter surfaces such as the forehead and chin tended to produce smaller deviations, while regions with more complex geometry showed greater variability across systems. However, these regional trends were not statistically significant.

These findings provided a controlled reference for understanding baseline performance differences among professional structured-light scanners, photogrammetry-based applications, and mobile LiDAR tools. Further studies incorporating diverse facial anatomies, mixed-effects statistical modeling, and in vivo scanning conditions are needed to evaluate how these systems perform in real-world clinical environments.

## Figures and Tables

**Table 1:** Summary of the Four 3D Facial Scanning Systems and Their Acquisition Parameters.

Scanning System	Technology	Camera and Resolution	Scanning Area	Scanning Distance	Lighting Conditions	Acquisition Procedure	Resolution/Output Quality
MetiSmile (Shining 3D, China)	Structured-light handheld scanner	3 data acquisition cameras (1.3 MP) + 1 HD texture camera (5.0 MP)	210 × 270 mm	40–50 cm	Neutral indoor illumination (~500 lux)	One continuous 360° sweep around the head, 15–20 s	Approx. 0.2 mm capture resolution
RAYFace v2.0 (Ray Co., Republic of Korea)	Desktop structured-light system	6 cameras (1440 × 1080 px)	220 × 300 mm	Fixed 50 cm	Controlled studio lighting, 5500 K, shadow-free	Automatic acquisition using 5 synchronized cameras; 3 composite scans merged by software	Effective resolution ~0.15 mm
Heges (iOS LiDAR app)	Smartphone-based LiDAR depth scanning	Dual camera (Main 48 MP)	~30–50 cm (depending on scanning distance)	30–40 cm	Diffuse ambient lighting	Three circumferential passes (frontal, right, left) to minimize occlusion	Approx. 0.3 mm depth resolution
Polycam (iOS photogrammetry mode)	Smartphone-based photogrammetry	iPhone camera (48 MP)	Not fixed (≥20 multi-angle images)	30–50 cm	Evenly distributed lighting (~5000 K)	80–120 overlapping images acquired around the head; photogrammetric 3D mesh reconstruction	Effective ~0.25 mm point-cloud resolution

**Table 2:** Comparison of Trueness and Precision among 3D Facial Scanning Systems.

Scanner System	Trueness (mm)	Precision (mm)
Polycam (PC)	0.49 ± 0.32	0.15 ± 0.06
MetiSmile (MS)	0.51 ± 0.36	0.12 ± 0.07
RAYFace (RF)	0.58 ± 0.39	0.28 ± 0.19
Heges (HG)	0.73 ± 0.42	0.41 ± 0.17
Caliper (Gold Standard)	-	0.02 ± 0.03

Note: Data were presented as mean ± standard deviation. Kruskal–Wallis test comparing trueness among RAYFace, MetiSmile, and Polycom yielded *p* = 0.148 (>0.05), indicating no statistically significant difference in trueness among these three systems. Regarding the precision, the one-way ANOVA test showed a statistically significant difference among the systems (*p* < 0.001). Post hoc Tukey comparisons revealed significant pairwise differences between RF–PC, MS–HG, MS–PC, and HG–PC (all *p* < 0.05).

**Table 3:** Comparison of Trueness and Precision among Five Facial Regions using Four 3D Facial Scanning Systems.

Facial Region	Trueness (mm)	Precision (mm)	*p*
Forehead	0.42 ± 0.29	0.22 ± 0.13	>0.05
Eyes	0.59 ± 0.34	0.26 ± 0.15	
Nose	0.66 ± 0.45	0.28 ± 0.16	
Cheeks	0.82 ± 0.41	0.24 ± 0.12	
Chin	0.51 ± 0.27	0.22 ± 0.12	

Note: Data were presented as mean ± standard deviation. One-way ANOVA was conducted to compare trueness and precision values among five facial regions (forehead, eyes, nose, cheeks, and chin) based on pooled data from four 3D facial scanning systems. No statistically significant differences were found among regions (*p* > 0.05), indicating consistent accuracy and repeatability across facial areas.

## Data Availability

The data presented in this study are available on request from the corresponding author. The data are not publicly available due to institutional policy and laboratory confidentiality.
